# Fetal exposure to a mixture of endocrine‐disrupting chemicals and biomarkers of male fecundity: A population‐based cohort study

**DOI:** 10.1111/andr.70039

**Published:** 2025-04-12

**Authors:** Sidsel Dan Hull, Karin Sørig Hougaard, Gunnar Toft, Kajsa Kirstine Ugelvig Petersen, Esben Meulengracht Flachs, Christian Lindh, Cecilia Høst Ramlau‐Hansen, Lauren A. Wise, Allen Wilcox, Zeyan Liew, Jens Peter Bonde, Sandra Søgaard Tøttenborg

**Affiliations:** ^1^ Department of Occupational and Environmental Medicine Copenhagen University Hospital ‐ Bispebjerg and Frederiksberg Copenhagen Denmark; ^2^ Department of Public Health University of Copenhagen Copenhagen Denmark; ^3^ National Research Center for the Working Environment Copenhagen Denmark; ^4^ Steno Diabetes Center Aarhus, Aarhus University Hospital Aarhus Denmark; ^5^ Division of Occupational and Environmental Medicine, Lund University Lund Sweden; ^6^ Department of Public Health, Aarhus University Aarhus Denmark; ^7^ Department of Epidemiology Boston University School of Public Health Boston Massachusetts USA; ^8^ National Institute of Environmental Health Sciences Durham North Carolina USA; ^9^ Department of Environmental Health Sciences Yale School of Public Health New Haven Connecticut USA

**Keywords:** chemical mixtures, early life exposures, endocrine‐disrupting chemicals (EDCs), environmental pollutants, reproductive hormones, semen quality

## Abstract

**Background:**

Fetal exposure to endocrine‐disrupting chemicals (EDCs) has been associated with reduced male fecundity, but with few studies considering chemical mixtures.

**Objectives:**

We assessed the association between fetal exposure to a mixture of EDCs and biomarkers of male fecundity in young adulthood.

**Materials and methods:**

The study population comprised 841 young adult males enrolled in the Fetal Programming of Semen Quality cohort, established as a male offspring sub‐cohort within the Danish National Birth Cohort. Maternal blood samples were analyzed for concentrations of per‐ and polyfluoroalkyl substances (PFAS), phthalate metabolites, and triclosan. We used quantile g‐computation to estimate the change in semen characteristics, testicular volume, and reproductive hormone levels with 95% confidence intervals (CI) per one‐quartile increase in all chemicals within three chemical mixtures; an overall chemical mixture, a PFAS mixture, and a non‐persistent chemical mixture.

**Results:**

Fetal exposure to a one‐quartile increase in the overall chemical mixture was associated with 4.0 million/mL lower sperm concentration (95% CI: −9.1, 1.1), 16.1 million lower total sperm count (95% CI: −33.8, 1.6), 0.5 mL smaller testicular volume (95% CI: −1.2, 0.3), 5% higher proportion of non‐progressive and immotile spermatozoa (95% CI: 0.99, 1.11), and 7% higher concentration of FSH (95% CI: 0.99, 1.16), but with limited precision. Effect sizes were greatest in magnitude for sperm concentration and total sperm count. We observed somewhat similar associations for the PFAS mixture and no associations for the non‐persistent chemical mixture.

**Discussion:**

Results suggest that fetal exposure to an overall mixture of EDCs may be adversely associated with several biomarkers of male fecundity, but findings are also compatible with null associations. These associations, if true, appeared to be driven by PFAS, but misclassification due to a single measurement of the phthalate metabolites and triclosan may have attenuated the results.

## 1 INTRODUCTION

Pregnant women are exposed to a multitude of chemicals through diet, drinking water, and consumer products, such as textiles, plastics, and cosmetics.^1^, ^2^ Additional exposure may occur in their work life.^3^, ^4^ Some of these chemicals possess endocrine‐disrupting properties and can alter functions of the endocrine system.^5^ These endocrine‐disrupting chemicals (EDCs) may cross the placenta, enter the fetal circulation, and accumulate in the developing fetus.^6^, ^7^, ^8^, ^9^, ^10^ Endocrine disruption during organogenesis can permanently alter organ function, also later in life.^11^ Concerns about fetal exposure to EDCs have prompted a large number of studies, with results implicating a variety of adverse health effects in the offspring, including male reproductive disorders.^12^, ^13^, ^14^, ^15^, ^16^, ^17^, ^18^

The human male reproductive organs develop primarily during the masculinization programming window within gestational weeks 7–15.^19^, ^20^ During this window the secretion of androgens, such as testosterone produced in the fetal testes, is important for normal male reproductive organ development.^20^ Thus, exposure to EDCs during the masculinization programming window may disturb normal development of the male reproductive organs.^21^

Although fetal exposure to EDCs is indicated to interfere with male reproductive function later in life, human evidence is inconsistent and based almost exclusively on studies evaluating EDCs individually without consideration of interaction or confounding by other EDCs.^13^, ^21^ If the additivity or synergism between chemicals is not considered, the total mixture effects may be underestimated. Further, not addressing multicollinearity of highly correlated chemicals may lead us to ascribe an effect to the wrong chemical.^22^, ^23^

Studies in rats demonstrate associations of fetal exposure to mixtures of EDCs with reproductive endpoints in male offspring.^24^, ^25^, ^26^, ^27^ Some of these studies further indicate that chemicals with common health outcomes may be assessed together even though their modes or mechanisms of action may be different.^25^, ^27^ In humans, a recent mixture risk assessment of 29 chemicals associated with deteriorations of semen quality showed that acceptable mixture exposure levels were exceeded by a large proportion within a population of young Danish males.^28^

Epidemiological studies of chemical mixtures are emerging. Cross‐sectional studies have shown some associations of exposure to different chemical mixtures of EDCs (including PFAS, phthalates, metals, organochlorines, parabens, phenols, and polycyclic aromatic hydrocarbons) and measures of adverse male fecundity.^29^, ^30^, ^31^, ^32^, ^33^, ^34^, ^35^, ^36^ However, only a few studies exist in the context of fetal exposure to mixtures of EDCs and male fecundity later in life.^37^, ^38^, ^39^, ^40^ A study embedded in the Generation R study in the Netherlands showed that fetal exposure to a mixture of bisphenols and phthalates was associated with earlier pubic hair development at 13 years, but not other reproductive developmental outcomes.^37^ Fetal exposure to a different mixture of EDCs (organochlorine compounds and polybrominated diphenyl ethers) was associated with shorter anogenital distance (a marker of reproductive toxicity) among Spanish boys aged 7–8 years,^39^ and a Chinese study showed that prenatal exposure to a mixture of PFAS was associated with longer anogenital distance in newborns.^38^

Our research group has previously observed that fetal exposure to a mixture of PFAS was associated with lower sperm concentration and total sperm count, as well as increased proportions of non‐progressive and immotile spermatozoa, among young Danish males.^40^ Failing to consider levels of other EDCs left us uncertain whether the observed association could be entirely ascribed to PFAS and if the effect therefore was underestimated. Using the same cohort of young Danish males, the primary aim of the present study was to repeat the assessment of fetal exposure to a mixture of PFAS, but in combination with two additional chemical classes of EDCs (i.e., phthalates and triclosan) on biomarkers of male fecundity in young adulthood.

## 2 MATERIALS AND METHODS

### 2.1 Study population

This study was based on the Fetal Programming of Semen Quality (FEPOS) cohort,^41^ established in 2017 as a sub‐cohort of young males born to mothers enrolled in the Danish National Birth Cohort (DNBC).^41^, ^42^ In total, 100,418 pregnancies were included in the DNBC from 1996 to 2002, resulting in a total of 49,653 live‐born sons. Sons were considered eligible for the FEPOS cohort, if they lived in relative proximity to the FEPOS clinic in Copenhagen or Aarhus and if their mothers provided a blood sample during pregnancy and responded to two computer‐assisted telephone interviews around gestational week 16 and 30. Sons who had undergone sterilization, cancer treatment, or orchidectomy were encouraged to decline participation. A total of 21,623 sons were eligible for inclusion and 5,697 were invited to participate as they turned 18 years and 9 months until we reached ∼1000 study participants. In total, 1,058 (participation rate: 19%) of the invited sons aged 18–20 years provided informed consent for participation, completed a questionnaire and a clinical examination, and provided semen, urine and blood samples between March 2017 and December 2019.^41^ We excluded participants with insufficient maternal plasma from the biobank for chemical analyses (*n* = 188), those who self‐reported having one or both testicles undescended (*n* = 6), and participants with missing data on potential confounders (*n* = 23). Accordingly, the final study population consisted of 841 young adult males. A flowchart of the selection of the study population is shown in Figure S1.

### 2.2 Semen characteristics and testicular volume

Semen samples were collected by masturbation with a recommended 2–3 days abstinence, but all samples were included regardless of abstinence time. Semen analyses were initiated as quickly as possible after collection (83% of the samples within 1 hour) by trained laboratory technicians (one at each clinic). Semen volume was measured by weight (1 gram = 1 milliliter [mL]) followed by a manual analysis of sperm concentration, total sperm count (sperm concentration × semen volume), and the proportions of progressive, non‐progressive and immotile spermatozoa. The proportion of morphologically normal spermatozoa and DNA fragmentation index (DFI) were analyzed at the Reproductive Medicine Centre, Skåne University Hospital, in Malmö, Sweden. DFI was analyzed by flow cytometry semen chromatin structure assay (SCSA®). All analyses, but DFI, followed the 2010 guidelines from the World Health Organization (WHO),^43^ and are described in details in Hærvig et al.^41^

Testicular volume was measured by participants themselves using a Prader orchidometer with 12 volumetric ellipsoids ranging from 1 to 25 mL.

### 2.3 Reproductive hormone levels

Levels of reproductive hormones were measured in non‐fasting plasma collected at the clinical examination and stored at −80° until analysis, which were performed by the Department of Biochemistry at Aarhus University Hospital, Aarhus, Denmark.^41^ Concentrations of testosterone and estradiol were measured using liquid chromatography–triple quadrupole linear ion trap mass spectrometry (LC–MS/MS). The limit of detection (LOD) was 0.12 nmol/L for testosterone and 15 pmol/L for estradiol. Follicle‐stimulation hormone (FSH), luteinizing hormone (LH), and sex hormone‐binding globulin (SHBG) were measured with immunoassays. The LOD was 0.1 IU/L for FSH and LH, and 0.35 nmol/L for SHBG.

### 2.4 Endocrine‐disrupting chemicals

Maternal plasma samples were collected during pregnancy (95% of which was collected during first trimester) and subsequently stored at −80°C in the Danish National Biobank. They were analyzed for chemicals in two separate batches between 2019 and 2021 by the Division of Occupational and Environmental Medicine at Lund University. Chemical concentrations were analyzed according to a modified method^44^, ^45^ using LC–MS/MS. A large part of the chemicals measured were not discernible or with few samples above the LOD. Our criterion for inclusion of individual chemical compounds in the statistical analyses was concentrations above the LOD in >75% of samples. Of 15 PFAS, ten phthalate metabolites, and triclosan analyzed, we included seven PFAS (perfluorohexanesulfonic acid [PFHxS], perfluoroheptanoic acid [PFHpA], perfluorooctanoic acid [PFOA], perfluorooctane sulfonic acid [PFOS], perfluorononanoic acid [PFNA], perfluorodecanoic acid [PFDA], perfluoroundecanoic acid [PFUnDA]), two phthalate metabolites (mono‐(2‐ethyl‐5‐carboxypentyl) phthalate [5cx‐MEPP] and mono‐(4‐methyl‐7‐carboxyheptyl) phthalate [cx‐MiNP]), and triclosan in our statistical analyses. Further details on measurement methods and chemicals not achieving the LOD threshold can be found in Text S1, Supporting Information. For chemical concentrations that were below the LOD, machine‐reported values by the LC–MS/MS were kept as is. In the statistical analyses all chemical concentrations were divided into quartiles and the values below the LOD ended up in the lowest quartile. The same chemicals were measured in young males’ plasma, but their concentrations of PFHpA and triclosan did not meet the criterion for inclusion in the analysis (<75% of the samples were above the LOD).

### 2.5 Covariates

Self‐reported information on maternal first‐trimester smoking, weekly alcohol intake, pre‐pregnancy body mass index (BMI), and the highest household occupational status in the family was available from the first DNBC telephone interview conducted around gestational week 16. Information on maternal age at birth and parity was obtained from the Danish Medical Birth Registry.^46^ At the clinical examination of the young males, we recorded their age, BMI, abstinence time in days, spillage, time from ejaculation to semen analysis, and time of day for blood sampling. Varicocoele, hypospadias, and cryptorchidism presence was self‐reported by history of diagnosis or previous treatment only. Smoking habits were also self‐reported. Categorizations of the covariates can be seen in Table 1.

### 2.6 Statistical analyses

Baseline characteristics were described through calculation of medians (with 5th and 95th percentiles) or by the number and percentage of individuals within a group. Plasma concentrations of chemicals were examined through calculations of medians and percentiles (5th, 25th, 75th, and 95th). All medians and percentiles were calculated as the mean of the five values nearest to the actual value to comply with local data regulation (GDPR, Regulation (EU), 2016/679 of 25 May 2018). Correlation patterns of chemical plasma concentrations were analyzed with Spearman's *ρ*.

To estimate the joint effect of fetal exposure to a mixture of EDCs on biomarkers of male fecundity, we used the quantile‐based g‐computation (qgcomp) approach.^47^ This approach estimates the effect of simultaneously increasing all exposures within a chemical mixture by one quartile. Qgcomp further allows estimation of the joint effect of a specific subset of compounds within the mixture (e.g., PFAS) while adjusting for possible confounding from other chemicals in the mixture (e.g., phthalate metabolites and triclosan). We estimated the effect of fetal exposure to three chemical mixtures: an overall chemical mixture (i.e., a mixture of seven PFAS, two phthalate metabolites, and triclosan), a PFAS mixture (i.e., a mixture of seven PFAS), and a non‐persistent chemical mixture (i.e., a mixture of two phthalate metabolites and triclosan). The PFAS mixture was adjusted for maternal concentrations of phthalate metabolites and triclosan, while the non‐persistent mixture was adjusted for PFAS concentrations.

Using the qgcomp approach, all exposures were first transformed into quartiles and then fitted to an underlying generalized linear model. As a number of outcomes did not show normality, we assessed model fit for several regression models, including linear models with a Gaussian distribution, with or without log‐transformations of the outcomes, and Poisson models. The best model fit was achieved with a linear model for all outcomes, though log‐transformation was needed in the analyses of semen volume, percentage of non‐progressive and immotile spermatozoa, DFI, FSH, LH, and SHBG to achieve a satisfying fit. Testicular volume was assessed as the average volume of both testicles, and we modelled the sum of non‐progressive and immotile spermatozoa instead of progressive sperm cells. We applied 200 bootstrap samples in all analyses. Outliers were identified through visually assessing QQ plots of residuals and a critical evaluation of the outliers in relation to reference values of the outcomes.

The psi (*ψ*) coefficient from the qgcomp models is interpreted as the mixture coefficient and represent the change in the outcome per one‐quartile increase in all chemicals within the mixture. For outcomes that were not log‐transformed, effect estimates were reported as mean difference per one‐quartile increase in all exposures in the mixture. For log‐transformed outcomes, effect estimates were reported as percent difference in the outcome per one‐quartile increase in all exposures in the mixture and was calculated using the following equation: ([exp(*ψ*) − 1] × 100).

We reported crude effect estimates and effect estimates adjusted for maternal age at birth, pre‐pregnancy BMI, first‐trimester smoking, weekly alcohol intake during pregnancy, and household occupational status identified a priori as potential confounders using existing literature and Directed Acyclic Graphs^48^ (Figure S2). To increase precision, we further adjusted analyses of semen characteristics for abstinence time, spillage (except for semen volume and total sperm count which were restricted to persons reporting no spillage), and minutes from ejaculation to sperm analysis (for the analysis of non‐progressive and immotile spermatozoa). Analyses for testicular volume was adjusted for abstinence time, while reproductive hormone levels were adjusted for time of day for blood sampling, and the young males' BMI. Finally, all analyses were adjusted for the batch number in the analyses of maternal concentrations of EDCs, and trimester of blood sampling. The number of individuals in each analysis depended on missing data of the outcome and the precision variables.

To explore non‐linear relationships between the chemical mixtures and the outcomes, we included a quadratic term for each chemical exposure within the mixture. The joint effect of the non‐linear analyses was determined by a quadratic term coefficient as well as the coefficient for the lower order joint effect, as for a traditional linear regression model. A significant quadratic term in the non‐linear analyses was considered an indication of non‐linearity of the mixture (*p* value <0.05). We did not include interaction terms in our models and thus did not formally evaluate non‐additive effects.

For the adjusted models of fetal exposure, we investigated which chemical concentrations within the overall chemical mixture were contributing the most to the observed effect estimates. Because the qgcomp package does not estimate weights when using bootstrapping, we examined weights as an exploratory sensitivity analyses using models similar to our primary models, but without bootstrapping. Weights from the qgcomp models can be interpreted as the relative contributions of each chemical to either the positive or negative partial effects. The weights should not be interpreted as individual effect sizes as models were performed to estimate joint effects of an overall chemical mixture.

Finally, we assessed associations between the study participants current chemical exposure and biomarkers of their fecundity. We assessed the same three chemical mixtures as assessed for fetal exposure, however, excluding PFHpA and triclosan as these chemicals did not meet our LOD criterion. Potential confounders in the analyses of the study participants current exposure to chemical mixtures included the study participants BMI and smoking status in young adulthood, maternal first‐trimester smoking, highest household occupational status in the family, and maternal levels of EDCs during pregnancy. Analyses were further adjusted for the same precision variables as the analyses of the fetal chemical mixtures.

Our approach to interpretation of data was based on an evaluation of the magnitude, direction, and precision of the effect estimates rather than binary significance testing. All statistical analyses were performed using R version 4.4.1 (RStudio 2021; PBC, Boston, MA, USA) and Stata version 18 (StataCorp, College Station, TX, USA).

## 3 RESULTS

Median maternal age at birth was 31 years (Table 1). Most mothers of participants were non‐smokers, did not drink alcohol, and 34% had a high household occupational status during the first trimester of pregnancy. The median age of study participants was 19 years. About half consumed alcohol on a weekly basis, 41% smoked, and 77% were enrolled at the Copenhagen clinic.

**TABLE 1:** Baseline characteristics of study participants and their mothers (*N* = 841).

	Median (5th–95th percentile)^a^ or *n* (%)
**Mothers**	
Age at birth of son (years)	31 (24–38)
Pre‐pregnancy BMI (kg/m^2^)	
Underweight (<18.5)	49 (6%)
Normal weight (18.5–25)	618 (73%)
Overweight (25–30)	137 (16%)
Obesity (≥30)	37 (4%)
Weekly alcohol consumption ≥1unit/week	389 (46%)
Smoking in first trimester	
Non‐smoker	651 (77%)
Light smoker (0–10 cigarettes/day)	164 (20%)
Heavy smoker (>10 cigarettes/day)	26 (3%)
Household occupational status	
High grade profession	282 (34%)
Low grade profession	280 (33%)
Skilled or unskilled worker	240 (29%)
Student or economically inactive	39 (5%)
Parity	
Primipara	365 (44%)
Multipara	460 (56%)
**Study participants**	
Age at clinical visit	19 (18–20)
BMI (kg/m^2^)	
Underweight (<18.5)	56 (7%)
Normal weight (18.5–25)	632 (75%)
Overweight (25–30)	130 (16%)
Obesity (≥30)	20 (2%)
Current of occational smoker (yes)	341 (41%)
Weekly alcohol consumption (yes)	442 (57%)
History of hypospadias or cryptorchidism (yes)^b^	26 (3%)
History of varicocoele (yes)^b^	8 (1%)
Sampling site	
Copenhagen clinic	646 (77%)
Aarhus clinic	195 (23%)
Abstinence time	
<2 days	288 (34%)
2–4 days	508 (61%)
≥5 days	40 (5%)
Spillage of ejaculate sample (yes)	142 (17%)
≤1 hour to sample analysis	696 (83%)
Time of day for blood sampling	
Morning (before 12 pm)	305 (37%)
Midday (between 12 and 6 pm)	439 (53%)
Evening (after 6 pm)	88 (11%)

Missing: parity (*n *= 16), study participants BMI (*n *< 5), study participants smoking habits (*n *< 5), study participants weekly alcohol consumption (*n *< 5), hypospadias or cryptorchidism (*n *< 5), abstinence time in days (*n *= 5), spillage of ejaculate sample (*n *= 8), time of day for blood sampling (*n* = 9).

Abbreviation: BMI, body mass index.

^a^ Due to local data regulations, median and percentiles were based on information from at least five individuals with values closest to the actual median/percentile.

^b^ Current or previous disorder.

Table 2 presents limits of detection for, and distributions of chemicals measured in maternal plasma samples during pregnancy. The median concentration of triclosan was 1.87 ng/mL and the highest median concentration of the phthalate metabolites was 0.76 ng/mL, reported for 5cx‐MEPP. Among the PFAS, PFOS and PFOA were measured at the highest median concentrations: 27.52 and 4.40 ng/mL, respectively. Triclosan was detectable with 85% of observed values >LOD. The included phthalate metabolites and PFAS were well detected with 100% of the observations >LOD, except for PFHpA, where 89% of the observations were >LOD.

**TABLE 2:** Limits of detection for and distributions of endocrine disrupting chemicals detected above the LOD in >75% of maternal plasma samples collected during pregnancy (*N* = 841).

	LOD (ng/mL)			Percentiles (ng/mL)^a^				
Chemical	Batch 1	Batch 2	%>LOD	5th	25th	50th	75th	95th
***Phenols***								
Triclosan	0.10	0.10	85	0.02	0.26	1.87	7.89	29.41
***Phthalate metabolites***								
5cx‐MEPP	0.05	0.02	100	0.31	0.51	0.76	1.11	1.99
cx‐MiNP	0.02	0.03	100	0.11	0.19	0.28	0.48	1.08
***PFAS***								
PFHxS	0.02	0.04	100	0.33	0.56	0.76	1.02	1.72
PFHpA	0.02	0.01	89	0.01	0.03	0.06	0.11	0.29
PFOA	0.02	0.03	100	2.03	3.23	4.40	5.87	8.29
PFOS	0.02	0.05	100	14.64	21.67	27.52	34.43	47.44
PFNA	0.02	0.01	100	0.20	0.30	0.38	0.48	0.69
PFDA	0.02	0.01	100	0.08	0.12	0.15	0.19	0.27
PFUnDA	0.02	0.01	100	0.06	0.09	0.12	0.16	0.22

*Note*: Other measured chemicals not quantified as the levels were not discernible or with <75% of samples above the LOD included: PFPeA, PFHxA, PFDoDA, PFTrDA, PFBS, PFPeS, PFHpS, PFDS, 2cx‐MEHP, 5‐oxo‐MEHP, 5‐OH‐MEHP, oxo‐MiNP, OH‐MiNP, OH‐MPHP, and MCiNP.

Abbreviations: 2cx‐MEHP, 2‐carboxymethyl hexyl phthalate; 5cx‐MEPP, mono‐(2‐ethyl‐5‐carboxypentyl) phthalate; 5‐OH‐MEHP, 5‐hydroxy mono(2‐ethylhexyl) phthalate; 5‐oxo‐MEHP, 5‐oxo mono(2‐ethylhexyl) phthalate; cx‐MiNP, mono‐(4‐methyl‐7‐carboxyheptyl) phthalate; LOD, limit of detection; MCiNP, mono(carboxyisooctyl) phthalate; OH‐MiNP, 7‐hydroxy monoisononyl phthalate; OH‐MPHP, hydroxy monoisopropyl phthalate; oxo‐MiNP, 7‐oxo monoisononyl phthalate; P, percentile; PFAS, per‐ and polyflouroalkyl substances; PFBS, perfluorobutanesulfonic acid; PFDA, perfluorodecanoic acid; PFDoDA, perfluorododecanoic acid; PFDS, perfluorodecane sulfonic acid; PFHpA, perfluoroheptanoic acid; PFHpS, perfluoroheptanesulfonic acid; PFHxA, perfluorohexanoic acid; PFHxS, perfluorohexane sulfonic acid; PFNA, perfluorononanoic acid; PFOA, perfluorooctanoic acid; PFOS, perfluorooctane sulfonic acid; PFPeA, perfluoropentanoic acid; PFPeS, perfluoropentanesulfonic acid; PFTrDA, perfluorotridecanoic acid.; PFUnDA, perfluoroundecanoic acid.

^a^ Due to local data regulations, median and percentiles were based on information from at least five individuals with values closest to the actual median/percentile.

The study participants current chemical concentrations were generally lower than their mothers, as the highest median concentration of the phthalate metabolites was 0.43 ng/mL, measured for cx‐MiNP, and the median concentration of PFOS and PFOA were 3.90 and 1.24 ng/mL, respectively (Table 3).

**TABLE 3:** Limits of detection for and distributions of endocrine disrupting chemicals detected above the LOD in >75% study participants plasma samples collected at enrollment in the FEPOS cohort (*N* = 827).

	LOD (ng/mL)			Percentiles (ng/mL)^a^				
Chemical	Batch 1	Batch 2	%>LOD	5th	25th	50th	75th	95th
***Phthalate metabolites***								
5cx‐MEPP	0.05	0.02	99	0.06	0.10	0.14	0.22	0.56
cx‐MiNP	0.02	0.01	100	0.16	0.30	0.43	0.67	3.39
***PFAS***								
PFHxS	0.02	0.04	100	0.17	0.25	0.32	0.41	0.60
PFOA	0.02	0.03	100	0.68	0.97	1.24	1.52	2.19
PFOS	0.02	0.05	100	2.10	3.11	3.90	5.17	8.18
PFNA	0.02	0.01	100	0.26	0.36	0.46	0.60	0.90
PFDA	0.02	0.01	100	0.10	0.14	0.18	0.24	0.34
PFUnDA	0.02	0.01	100	0.03	0.07	0.10	0.14	0.24

Abbreviations: 2cx‐MEHP, 2‐carboxymethyl hexyl phthalate; 5cx‐MEPP, mono‐(2‐ethyl‐5‐carboxypentyl) phthalate; 5‐OH‐MEHP, 5‐hydroxy mono(2‐ethylhexyl) phthalate; 5‐oxo‐MEHP, 5‐oxo mono(2‐ethylhexyl) phthalate; cx‐MiNP, mono‐(4‐methyl‐7‐carboxyheptyl) phthalate; FEPOS, Fetal Programming of Semen Quality; LOD, limit of detection; MCiNP, mono(carboxyisooctyl) phthalate; OH‐MiNP, 7‐hydroxy monoisononyl phthalate; OH‐MPHP, hydroxy monoisopropyl phthalate; oxo‐MiNP, 7‐oxo monoisononyl phthalate; P, percentile; PFAS, per‐ and polyflouroalkyl substances; PFBS, perfluorobutanesulfonic acid; PFDA, perfluorodecanoic acid; PFDoDA, perfluorododecanoic acid; PFDS, perfluorodecane sulfonic acid; PFHpA, perfluoroheptanoic acid; PFHpS, perfluoroheptanesulfonic acid; PFHxA, perfluorohexanoic acid; PFHxS, perfluorohexane sulfonic acid; PFNA, perfluorononanoic acid; PFOA, perfluorooctanoic acid; PFOS, perfluorooctane sulfonic acid; PFPeA, perfluoropentanoic acid; PFPeS, perfluoropentanesulfonic acid; PFTrDA, perfluorotridecanoic acid.; PFUnDA, perfluoroundecanoic acid.

^a^ Due to local data regulations, median and percentiles were based on information from at least five individuals with values closest to the actual median/percentile.

**FIGURE 1:**
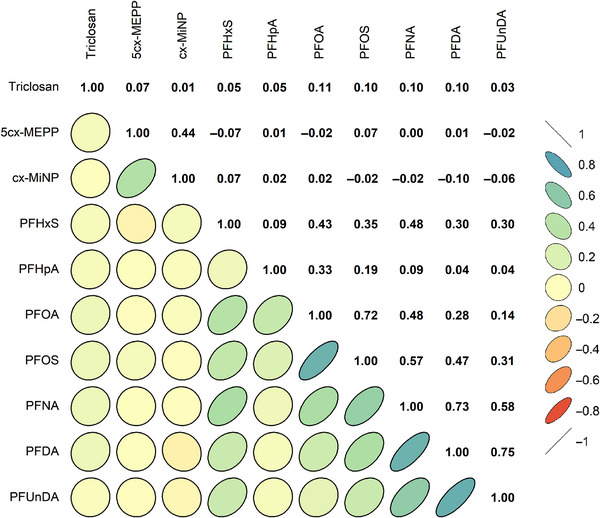
Spearman correlation matrix of endocrine disrupting chemicals measured in maternal plasma samples during pregnancy (*N* = 841). 5cx‐MEPP, mono‐(2‐ethyl‐5‐carboxypentyl) phthalate; cx‐MiNP, mono‐(4‐methyl‐7‐carboxyheptyl) phthalate; PFHxS, perfluorohexane sulfonic acid; PFHpA, perfluoroheptanoic acid; PFOA, perfluorooctanoic acid; PFOS, perfluorooctane sulfonic acid; PFNA, perfluorononanoic acid; PFDA, perfluorodecanoic acid; PFUnDA, perfluoroundecanoic acid.

In maternal plasma samples, correlations of chemicals were positive and generally high within the chemical class of PFAS (e.g., Spearman correlation between PFOS and PFOA = 0.72), while correlations within phthalate metabolites were moderate (Spearman correlation between 5cx‐MEPP and cx‐MiNP = 0.44) (Figure 1). Correlations between chemical classes were lower in magnitude (Spearman correlations ≤0.11). Triclosan correlated negligibly with phthalate metabolites and PFAS compounds, and the phthalate metabolites had negligible or weak inverse correlations with PFAS compounds.

Correlations between maternal plasma concentrations and the study participants current plasma concentrations were weak for PFAS (Spearman correlations between 0.06 and 0.26) and negligible for phthalate metabolites and triclosan (Table 4).

**TABLE 4:** Spearman correlations of plasma concentrations of endocrine disrupting chemicals among study participants in the FEPOS cohort and their mothers (*N* = 827).

Chemical	Spearman's *ρ* (95% CI)
***Phthalate metabolites***	
5cx‐MEPP	−0.00 (−0.07, 0.07)
cx‐MiNP	0.06 (−0.01, 0.13)
***PFAS***	
PFHxS	0.16 (0.09, 0.22)
PFOA	0.18 (0.11, 0.25)
PFOS	0.06 (−0.00, 0.13)
PFNA	0.14 (0.07, 0.21)
PFDA	0.13 (0.06, 0.19)
PFUnDA	0.26 (0.19, 0.32)

*Note*: Chemical concentrations were available for 827 study participants.

See Table 2 for chemical abbreviations.

Crude and adjusted estimates for the associations between fetal exposure to chemical mixtures and biomarkers of male fecundity in young adulthood are shown in Table 5.  Adjusted models indicated that a one‐quartile increase in fetal exposure to the overall chemical mixture was associated with 4.0 million/mL lower sperm concentration (95% CI: −9.1 million/mL, 1.1 million/mL), 16.1 million lower total sperm count (95% CI: −33.8 million, 1.6 million), 0.5 mL smaller testicular volume (95% CI: −1.2 mL, 0.3 mL), 5% higher proportion of non‐progressive and immotile spermatozoa (95% CI: −1%, 11%), and 7% higher concentration of FSH (95% CI: −1%, 16%), but with limited precision. Effect sizes were greatest in magnitude for sperm concentration and total sperm count. Our results did not indicate associations between fetal exposure to the overall chemical mixture and semen volume, the percentage of morphologically normal spermatozoa, DFI, testosterone, estradiol, SHBG, or LH. Across all outcomes, crude and adjusted models were comparable.

**TABLE 5:** Crude and adjusted change (95% confidence intervals) in biomarkers of male fecundity per one‐quartile increase in all exposures within an overall chemical mixture, a PFAS mixture, and a non‐persistent chemical mixture measured in maternal plasma samples during pregnancy and estimated using linear models in quantile g‐computation.

			Overall mixture		PFAS mixture		Non‐persistent chemical mixture	

Biomarkers of male fecundity	*n*	Median (5th–95th P)^a^	Crude	Adjusted	Crude	Adjusted	Crude	Adjusted
					**Mean difference (*ψ*) (95% CI)**			
Sperm concentration (×10^6^ per mL)	829	39 (2–136)	−2.7 (−7.9, 2.6)	−4.0 (−9.1, 1.1)	−2.8 (−7.1, 1.5)	−4.1 (−8.3, 0.0)	0.2 (−3.4, 3.9)	0.2 (−3.5, 3.9)
Total sperm count (×10^6^)	688	104 (8–407)	−10.2 (−29.2, 8.7)	−16.1 (−33.8, 1.6)	−9.0 (−23.6, 5.7)	−15.3 (−29.5, −1.0)	−1.7 (−13.2, 9.8)	−0.6 (−11.4, 10.2)
Morphologically normal spermatozoa (%)	813	6 (0–15)	−0.3 (−0.9, 0.3)	−0.3 (−1.0, 0.3)	−0.3 (−0.7, 0.1)	−0.4 (−0.8, 0.1)	0.0 (−0.5, 0.4)	0.0 (−0.5, 0.5)
Average testicular volume (mL)	836	15 (7–25)	−0.5 (−1.2, 0.3)	−0.5 (−1.2, 0.3)	−0.2 (−0.8, 0.3)	−0.2 (−0.7, 0.3)	−0.3 (−0.8, 0.3)	−0.2 (−0.7, 0.3)
Testosterone (nmol/L)	828	18 (10–29)	0.4 (−0.3, 1.0)	0.3 (−0.4, 0.9)	−0.1 (−0.7, 0.4)	−0.3 (−0.8, 0.2)	0.5 (0.0, 1.0)	0.6 (0.2, 1.1)
Estradiol (pmol/L)	828	53 (11–107)	−1.8 (−6.0, 2.4)	−1.2 (−5.3, 3.0)	−1.9 (−4.7, 0.9)	−1.5 (−4.2, 1.3)	0.2 (−2.7, 3.1)	0.3 (−2.5, 3.1)
					**Percent (%) difference (95% CI)**			
Semen volume (mL)^*^	688	3 (1–5)	−1 (−8, 8)	−3 (−10, 5)	−1 (−7, 5)	−3 (−9, 3)	0 (−5, 6)	1 (−5, 6)
Non‐progressive and immotile spermatozoa (%)^*^	813	36 (16–70)	5 (−1, 11)	5 (−1, 11)	7 (2, 11)	8 (3, 12)	−2 (−6, 2)	−3 (−7, 1)
DNA fragmentation index (%)^*^	776	10 (4–21)	−2 (−9, 6)	−2 (−9, 6)	1 (−5, 7)	0 (−6, 5)	−3 (−8, 2)	−2 (−7, 3)
FSH (IU/L)^*^	827	4 (1–8)	7 (−1, 16)	7 (−1, 16)	6 (0, 12)	6 (1, 12)	1 (−4, 6)	1 (−4, 7)
LH (IU/L)^*^	827	5 (3–9)	1 (−4, 7)	1 (−4, 7)	3 (−1, 7)	4 (0, 8)	−2 (−6, 2)	−2 (−6, 2)
SHBG (nmol/L)^*^	828	33 (17–58)	1 (−4, 5)	0 (−4, 4)	−1 (−4, 3)	−2 (−5, 1)	1 (−2, 5)	2 (−1, 6)

Psi (*ψ*) is the mixture coefficient and represent the change in biomarkers of male fecundity.

Asterisk (*) indicates models where outcomes were natural log (ln)‐transformed; effect estimates thus reflect percentage difference in the outcome per quartile increase in the mixture (calculated using the equation: ([exp(*ψ*) − 1] × 100).

Adjusted models included covariates for maternal age at birth, pre‐pregnancy BMI, maternal smoking, maternal alcohol consumption, and family occupational status during pregnancy, batch for analyses of maternal concentrations of endocrine disrupting chemicals, and trimester of blood sampling. Semen characteristics were also adjusted for abstinence time, spillage (except for semen volume and total sperm count which were restricted to persons reporting no spillage), and time from ejaculation to analysis (only for the analysis of non‐progressive and immotile spermatozoa). Testicular volume was adjusted for abstinence time, while reproductive hormone levels were adjusted for time of day for blood sampling and the young males BMI. The PFAS mixture was further adjusted for maternal concentrations of triclosan and phthalate metabolites during pregnancy and the non‐persistent chemical mixture was adjusted for maternal concentrations of PFAS. Abbreviations: P, percentile; CI, confidence interval; PFAS, per‐ and polyfluoroalkyl substances; FSH, follicle stimulating hormone; LH, luteinizing hormone; SHBG, sex hormone binding globulin.

^a^ Due to local data regulations, median and percentiles were based on information from at least five individuals with values closest to the actual median/percentile.

We observed somewhat similar associations for the PFAS mixture when adjusting for the non‐persistent chemicals (Table 5). For example, a one‐quartile increase in fetal exposure to the PFAS mixture was associated with lower sperm concentration (−4.1 million/mL, 95% CI: −8.3, 0.0) and lower total sperm count (−15.3 million, 95% CI: −29.5, −1.0). The PFAS mixture was also associated with 4% higher concentration of LH (95% CI: 0%, 8%), but with limited precision. We observed no associations for the non‐persistent chemical mixture when adjusting for PFAS.

Our exploratory analyses of the adjusted models of fetal exposure to different chemical mixtures and biomarkers of male fecundity including non‐linear (i.e., quadratic) terms for all exposures did not indicate non‐linearity of any of the chemical mixtures (Table S1).

Although we could not estimate weights from our bootstrapped models, we estimated weights for the adjusted models of the overall chemical mixture without bootstrapping as a sensitivity analysis (Table S2). Results from these models were overall comparable with the results from the bootstrapped models. The estimated weights indicated that PFAS contributed most strongly to the observed associations for the overall chemical mixture. For example, PFHpA had the highest negative contribution to the indicated association with sperm concentration and PFDA had the highest negative contribution for total sperm count.

Finally, we investigated associations between study participants current chemical exposure in relation to their fecundity (Table 6). These models indicated that a one‐quartile increase in current exposure to all chemicals in the overall chemical mixture was associated with 4% higher FSH (95% CI: ‐3%, 11%), and 4% higher SHBG (95% CI: 0%, 8%), but with limited precision. No associations were observed for semen characteristics or testicular volume. We observed similar associations for the PFAS mixture, but all null associations for the non‐persistent chemical mixture.

**TABLE 6:** Crude and adjusted change (95% confidence intervals) in biomarkers of male fecundity per one‐quartile increase in all exposures within an overall chemical mixture, a PFAS mixture, and a non‐persistent chemical mixture measured in study participants plasma samples collected at approximately age 19 years and estimated using linear models in quantile g‐computation.

		Overall mixture		PFAS mixture		Non‐persistent chemical mixture	
Biomarkers of male fecundity	*n*	Crude	Adjusted	Crude	Adjusted	Crude	Adjusted
				**Mean difference (*ψ*) (95% CI)**			
Sperm concentration (×10^6^ per mL)	816	1.5 (−3.1, 6.1)	1.8 (−2.9, 6.4)	−0.2 (−3.8, 3.4)	0.2 (−3.5, 3.8)	1.7 (−1.3, 4.7)	1.9 (−1.2, 4.9)
Total sperm count (×10^6^)	677	7.0 (−9.1, 23.1)	7.2 (−7.5, 22.0)	2.2 (−10.6, 15.0)	3.9 (−8.1, 16.0)	4.6 (−5.1, 14.2)	3.4 (−6.4, 13.2)
Morphologically normal spermatozoa (%)	801	0.2 (−0.3, 0.8)	0.0 (−0.5, 0.6)	−0.1 (−0.5, 0.4)	−0.2 (−0.6, 0.3)	0.3 (−0.1, 0.6)	0.2 (‐0.2, 0.5)
Average testicular volume (mL)	823	−0.2 (−0.8, 0.4)	0.2 (−0.5, 0.9)	−0.1 (−0.6, 0.4)	0.3 (−0.2, 0.8)	−0.2 (−0.7, 0.2)	−0.1 (−0.5, 0.3)
Testosterone (nmol/L)	825	−0.3 (−0.9, 0.3)	−0.3 (−0.9, 0.2)	0.2 (−0.3, 0.8)	0.2 (−0.2, 0.7)	−0.6 (−1.0, −0.2)	−0.6 (−1.0, −0.2)
Estradiol (pmol/L)	825	−0.8 (−3.8, 2.3)	−1.9 (−5.2, 1.3)	0.3 (−2.4, 3.0)	−0.3 (−3.2, 2.6)	−0.6 (−2.6, 1.4)	−1.6 (−3.6, 0.3)
				**Percent (%) difference (95% CI)**			
Semen volume (mL)^*^	677	0 (−6, 6)	−1 (−7, 5)	2 (−3, 7)	1 (−4, 6)	−2 (−6, 1)	−3 (−7, 1)
Non‐progressive and immotile spermatozoa (%)^*^	801	−4 (−8, 1)	−2 (−6, 2)	−3 (−6, 1)	−2 (−5, 1)	−1 (−4, 2)	0 (−3, 3)
DNA fragmentation index (%)^*^	764	5 (−1, 12)	1 (−5, 7)	3 (−2, 8)	0 (−4, 5)	2 (−2, 6)	0 (−4, 5)
FSH (IU/L)^*^	824	6 (−1, 12)	4 (−3, 11)	6 (1, 11)	5 (0, 10)	0 (−4, 4)	−1 (−5, 4)
LH (IU/L)^*^	824	2 (−2, 6)	1 (−3, 6)	0 (−3, 4)	0 (‐3, 3)	2 (−1, 4)	2 (−1, 4)
SHBG (nmol/L)^*^	825	3 (−2, 8)	4 (0, 8)	3 (−1, 6)	3 (0, 7)	0 (−3, 3)	0 (−2, 3)

Psi (*ψ*) is the mixture coefficient and represent the difference in biomarkers of male fecundity.

Asterisk (*) indicates models where outcomes were natural log (ln)‐transformed; effect estimates thus reflect percentage difference in the outcome per quartile increase in the mixture (calculated using the equation: ([exp(*ψ*) − 1] × 100).

Adjusted models included covariates for the young males BMI, smoking status, maternal smoking and household occupational status during pregnancy, and batch for analyses of the young males’ current concentrations of endocrine disrupting chemicals. Semen characteristics were also adjusted for abstinence time, spillage (except for semen volume and total sperm count which were restricted to persons reporting no spillage), and time from ejaculation to analysis (only for the analysis of non‐progressive and immotile spermatozoa). Testicular volume was adjusted for abstinence time, while reproductive hormone levels were adjusted for time of day for blood sampling. The PFAS mixture was further adjusted for study participants concentrations of triclosan and phthalate metabolites and the non‐persistent chemical mixture was adjusted for the study participants concentrations of PFAS.

Abbreviations: CI, confidence interval; FSH, follicle stimulating hormone; LH, luteinizing hormone; P, percentile; PFAS, per‐ and polyfluoroalkyl substances; SHBG, sex hormone binding globulin.

## 4 DISCUSSION

In this population‐based cohort study, our results indicated that higher fetal exposure to a mixture of seven PFAS, two phthalate metabolites, and triclosan was associated with lower sperm concentration, lower total sperm count, decreased testicular volume, higher proportion of non‐progressive and immotile spermatozoa, and higher concentration of FSH (a marker of primary testicular failure), but with limited precision. These associations were mainly driven by PFAS and were most notable for sperm concentration and total sperm count. The study participants current exposure to the overall chemical mixture did not show consistent associations.

In our original study based on the same data, fetal exposure to a mixture of PFAS was associated with lower sperm concentration, lower total sperm count, and a higher proportion of non‐progressive and immotile spermatozoa in young adulthood, using the weighted quantile sum (WQS) regression model.^40^ Despite the different statistical methods used, these findings were consistent with those presented herein. In a cross‐sectional study of the young adult males, also utilizing data from the FEPOS cohort, we observed no consistent associations between a mixture of PFAS and semen quality or testicular volume using the WQS regression model though higher concentrations of a PFAS mixture was associated with slightly higher levels of FSH,^31^ similar to the present study results. Our findings thus seem robust regardless of the statistical method applied.

To our knowledge, this is the first study to prospectively assess fetal exposure to a mixture of more than one group of EDCs in relation to biomarkers of male fecundity in young adulthood, hindering comparison of our results to others. However, a few single‐substance studies support associations between PFAS and phthalate metabolites and biomarkers of male fecundity in young adulthood.^45^, ^49^, ^50^, ^51^ A Danish study observed trends that higher in utero exposure to PFOA, but not PFOS, was associated with lower sperm concentration and total sperm count, as well as higher LH and FSH levels among young adult males.^49^ Contrary to our findings, studies on prenatal phthalate exposure have shown associations between different phthalate metabolites (e.g., mono‐isononyl phthalate [MiNP] and mono‐(2‐ethylhexyl) phthalate [MEHP]) and higher levels of FSH and LH, decreased testicular volume, lower total serum testosterone, and lower semen volume.^45^, ^50^, ^51^ Notably, the strongest associations were observed for the sum of phthalate metabolites in some of these studies.^50^, ^51^ A possible explanation for the conflicting results may be differences in the methods applied, exposure assessment of the non‐persistent chemicals, or varying exposure patterns across time and study populations.

Evidence from toxicological studies support the biologic plausibility of an association between fetal exposure to chemical mixtures of EDCs and biomarkers of male fecundity in young adulthood. One study in rats demonstrated that fetal exposure to a mixture of 13 EDCs (including phthalates, pesticides, UV‐filters, bisphenol A, parabens, and the drug paracetamol), at dose levels reflecting high‐end human intakes, can affect male sexual development.^24^ Another rat study demonstrated that perinatal exposure to a mixture of bisphenol A, butylparaben, di‐2‐ethylhexyl phthalate, and procymidone, that is, chemicals with diverse endocrine modes of action, was associated with reduced sperm counts and anogenital distance, and increased nipple retention, all markers of endocrine disruption, and hence, potentially, of disruption of male reproduction.^25^ Adverse male reproductive effects were also observed in offspring of rats exposed to a mixture of nine phthalates and five pesticides during the critical window of fetal masculinization (gestation day 14–18 in rats).^26^ Overall, these findings indicate that fetal exposure to a mixture of EDCs may, in concert, affect male offspring fecundity even though the modes or mechanisms of action may not be similar. PFAS, phthalates, and triclosan represents different modes or mechanisms of action, even within the chemical classes.^52^, ^53^, ^54^, ^55^ For instance, these chemicals can interfere both on a superior level via output and regulation of the hypothalamus–pituitary–gonadal axis, but also through interaction with nuclear and membrane bound receptors and cytosolic targets and alter function of critical steroidogenic enzymes.^52^, ^53^, ^54^, ^55^

Most effect sizes observed in this study were small to moderate and need to be carefully interpreted. The median sperm concentration in this study was 39 million/mL (5th–95th percentile: 2–136) and our results suggested that a one‐quartile increase in the overall chemical mixture was associated with 4.0 million/mL lower sperm concentration (95% CI: −9.1 million/mL, 1.1 million/mL). Such a reduction would result in more males having a sperm concentration close to the lower limit of the normal range which is 15 million/mL according to the WHO.^43^ Since low sperm concentration has been associated with low likelihood of pregnancy,^56^ our findings are thus considered relevant on a population level.

A core study strength of this study is the prospective assessment of concurrent exposure to several classes of EDCs. Since individuals are exposed to a wide range of chemicals every day, single‐substance assessments may fail to consider additive, synergistic, or antagonistic effects trough co‐exposure of chemicals. To move the field forward, methods such as Bayesian kernel machine regression (BKMR),^57^ WQS,^58^ and qgcomp^47^ are used increasingly to study chemical mixtures, but with no current consensus on which method to use in epidemiological studies of chemical mixtures, as different research questions may require different statistical methods.^23^, ^59^ We used qgcomp as we aimed to estimate the joint effect of a mixture of EDCs on biomarkers of male fecundity. This method provides simplicity of inference (in contrast to the BKMR), allows us to explore non‐linearity of mixture‐effect relationships, and does not have assumptions on directional homogeneity as weights can go in either a positive or negative direction (in contrast to the WQS where the direction of effect is assumed to be similar for all chemicals).^47^ The latter is important in the study of EDCs, as the precise mechanisms and direction of effects cannot always be predicted.^60^

Some other methodological considerations should also be noted. As in all epidemiological studies of chemical exposures, only a fraction of the chemicals is measured, usually due to financial or laboratory constraints. The effects observed for the specific mixtures studied could in theory be influenced by several other unknown or unmeasured chemicals. For example, unmeasured chemicals (e.g., bisphenols or replacement chemicals) that might correlate highly with levels of PFAS, phthalates, or triclosan and are also associated with biomarkers of male fecundity could introduce copollutant confounding or effect modification. Although we adjusted for several potential confounders, we cannot rule out the possibility of residual confounding by coexposures or unknown factors.

The chemical mixture assessed in the present study reflects exposure levels of PFAS, phthalates, and triclosan in the late 1990s as well as in the period 2017–2019. Since chemical mixtures are complex, their effects are often difficult to compare across studies because of variability in the prevalence and absolute levels of exposures across populations. For example, PFAS measured in the Danish population from 1988 until 2021 show a notable decrease following regulation and phase‐out of PFOS and PFOA.^61^ A similar decreasing trend has been observed for phthalates and triclosan in the Danish population, also most likely due to political regulation.^62^ During the same period, many replacement PFAS and phthalates have emerged and may increasingly be present in human populations.^62^, ^63^, ^64^ The exposure pattern of the studied EDCs has thus changed markedly during the last decades. These changes were also apparent in our study population as the study participants current chemical concentrations were much lower than their mothers. In fact, these differences in exposure levels may explain why we did not observe associations between study participants current chemical mixtures and semen characteristics. The generalizability of this study is thus limited due to the fraction of EDCs included in the study, and because of changes in the chemical body burden over time. Concentrations of the EDCs assessed in this study is, however, somewhat comparable with that of the same EDCs measured at the same time in other European countries,^44^ which increase the generalizability.

A considerable strength is the size of our study, which was based on the world's largest population‐based male offspring cohort with detailed information on biomarkers of male fecundity and extensive prospectively collected information on fetal and adult exposures.^41^ Despite the rather low participation rate (19%), selection bias due to self‐selection into the FEPOS cohort is of limited concern as study participants were aged 18–20 years and probably unaware of any fertility problems.^41^ Study participants were also unaware of their mothers’ concentrations of EDCs during pregnancy, and it is therefore unlikely that their participation depended on the exposure status, which limits the risk of selection bias.

Another strength of this study is that biomarkers of male fecundity were measured directly in biological samples using state‐of‐the‐art techniques by trained laboratory technicians blinded to the participants’ exposure status. Error from misclassification of the outcome is thus considered low, and if any, the misclassification is most likely non‐differential and independent of fetal exposure to EDCs. Nevertheless, all outcomes were assessed based on single samples which may lead to imprecise results because of the intraindividual variability of semen characteristics and diurnal cycles in the secretion of many reproductive hormones. To account for this, we included several precision variables in our adjusted analyses. Using more than one semen and plasma sample could have reduced potential measurement error, but asking for more than one sample could also have reduced participation and increased the risk of selection bias. Testicular volume was measured with an orchidometer by the participants themselves, a method previously described to obtain valid measurements of testicular volume.^65^ As participants were unaware of their exposure status, potential bias from under‐ or overestimation of their testicular volume would generally attenuate results.

All EDCs were measured using a validated LC–MS/MS method^44^ in single non‐fasting plasma samples at a laboratory successfully participating in interlaboratory controls. Given the long biological half‐life of PFAS in humans, we assumed that one measurement of PFAS reflected the average PFAS concentrations during the first trimester well. However, as PFAS can cross the placenta and accumulate in the developing fetus,^6^, ^7^, ^8^, ^9^, ^10^ maternal plasma samples collected during the second or third trimester (5% of samples) may not as accurately capture PFAS exposure during the first trimester when the male reproductive organs develop. Since most maternal plasma samples were collected during first trimester, we expect minimal misclassification of PFAS exposure. Due to the relatively short half‐lives of non‐persistent chemicals, a single measurement of phthalate metabolites and triclosan may not fully capture the individual variation in concentrations throughout pregnancy. However, given that many non‐persistent chemicals are ubiquitous, with exposure occurring frequently or even daily, a single maternal plasma sample in first trimester can still reflect exposure within the shorter masculinization programming window, before major pregnancy‐related changes such as increased blood volume occur. Some studies further suggest that a single measure of phthalates may be able to reasonable predict exposure categories in up to several months.^66^ Categorizing exposure biomarkers into quartiles, as we did, may thus reasonably reflect true exposure. One measurement during first trimester may be less indicative for later gestational exposure or early postnatal exposure, that needs to be fully examined. Studies that utilize at least two measurements of these non‐persistent chemicals collected during first trimester are also warranted. Another limitation is the use of plasma concentrations even though urinary measurements of phthalate metabolites and triclosan are often preferred. Since we utilized historical cohort biobank data from the DNBC, our only option was to measure these chemicals in plasma samples even though it may have introduced bias. Some phthalate metabolites measured in urine samples do, however, correlate with measurements in blood (including 5cx‐MEPP).^45^ We expect any misclassification of the triclosan and phthalate metabolites to be non‐differential because the assessment of the exposure and the outcomes were performed independent of each other. Such misclassification would generally attenuate associations and reduce precision, which could explain why we observed stronger signals for the PFAS mixture than for the overall chemical mixture which included triclosan and phthalate metabolites.

Some statistical considerations should also be noted. We explored non‐linearity of the chemical mixtures by including quadratic terms of each chemical exposure within the mixture. The evaluation of a non‐linear model was, however, exploratory, and may have been limited by statistical power and should not be relied upon for robust inference. Furthermore, some biomarkers of male fecundity (i.e., sperm concentration, total sperm count, morphologically normal spermatozoa, testosterone, estradiol and average testicular volume) are better modeled with negative binomial distribution, but the qgcomp package in R did not allow us to specify a negative binomial distribution and our findings should therefore be interpreted with caution as this may have caused bias in either direction, even though our findings were based on the best model fit available. We did, however, observe findings comparable to those of our previous study using the WQS regression with a negative binomial distribution, indicating that our results are somewhat robust regardless of the distributional assumption of the statistical models.

In conclusion, our results indicated that higher fetal exposure to a chemical mixture of seven PFAS, two phthalate metabolites, and triclosan was associated with lower sperm concentration and total sperm count, decreased testicular volume, higher proportion of non‐progressive and immotile spermatozoa, and higher concentration of FSH (a marker of primary testicular failure), but with limited precision. We observed somewhat similar associations for a PFAS mixture and no associations for a non‐persistent chemical mixture. Associations, if true, appeared to be driven by PFAS, but misclassification due to a single measurement of the phthalates and triclosan may have attenuated results. Current exposure to a chemical mixture of PFAS and phthalate metabolites was associated with higher levels of FSH and SHBG, but with limited precision, and was not associated with other reproductive hormones, semen characteristics, or testicular volume. Studies with repeated measurements of non‐persistent chemicals, are needed to expand our knowledge on the endocrine‐disrupting effects on male fecundity.

## AUTHOR CONTRIBUTIONS

Sandra Søgaard Tøttenborg, Jens Peter Bonde, Karin Sørig Hougaard, Cecilia Høst Ramlau‐Hansen, Gunnar Toft, and Kajsa Ugelvig Petersen contributed to the conception of the study. Sandra Søgaard Tøttenborg and Jens Peter Bonde acquired funding for the FEPOS cohort. The data collection in FEPOS was planned and headed by Sandra Søgaard Tøttenborg, Jens Peter Bonde, Karin Sørig Hougaard, Cecilia Høst Ramlau‐Hansen, and Gunnar Toft. Christian Lindh performed chemical analyses. Sandra Søgaard Tøttenborg and Sidsel Dan Hull administered the project. Sidsel Dan Hull performed data management, statistical analysis, and wrote the first draft of the manuscript. All authors made substantial contributions to the methodology of the study, interpretation of results, revision of the article, and approved the final version to be published.

## CONFLICT OF INTEREST STATEMENT

Lauren Wise is a paid consultant for Abbvie Inc. and the Gates Foundation for her work unrelated to the topic of this manuscript. Lauren Wise receives in‐kind donations from Kindara.com. The remaining authors declare that they have no known competing financial interests or personal relationships that could have appeared to influence the work reported in this paper.

## Supporting information



Supporting Information

## Data Availability

The data analyzed in the study are not publicly available due to national data security legislation on sensitive personal data. Researchers may apply for access to data from the Danish National Birth Cohort. Please see https://www.dnbc.dk/data‐available for additional information.
