# Supplementary material for: Fetal exposure to a mixture of endocrine‐disrupting chemicals and biomarkers of male fecundity: A population‐based cohort study
Source: Andrology. 2025 Apr 12;14(1):76–89. doi: 10.1111/andr.70039 (PMC12670469; doi:10.1111/andr.70039)
Supplement: Supplementary file 1 — Supporting Information [file ANDR-14-76-s001.docx]

**Supplementary materials**

**Supplemental text**

**Text S1.** Details on the analytical method used to measure chemical concentrations.

**Supplemental figures**

**Figure S1.** Flowchart of the selection of the study population.

**Figure S2.** Directed Acyclic Graph of the assumed relations between fetal exposure to a mixture of endocrine disrupting chemicals and biomarkers of male fecundity in young adulthood.

**Supplemental tables**

**Table S1.** Adjusted mixture coefficients and quadratic terms (95% confidence intervals) for associations between biomarkers of male fecundity and chemical mixtures measured in maternal plasma samples during pregnancy.

**Table S2.** Adjusted change (95% confidence intervals) in biomarkers of male fecundity per one-quartile increase in all exposures within the overall chemical mixture and weights representing the proportion of positive or negative partial effects for each exposure biomarker using linear models in quantile g-computation models without bootstrapping.

**Text S1.** Details on the analytical method used to measure chemical concentrations.

Quality control samples, chemical blanks, and calibration standards were included in the analysis of each sample batch (96-well plate). The LOD was defined as three times the standard deviation of the concentrations in chemical blank samples (**Table 2**).

Our criterion for inclusion of compounds in the statistical analyses was concentrations detectable above the limit of detection (LOD) in >75% of the samples. In the analytical methods several additional compounds were included, but not quantified as the levels were not discernible or too few above LOD were not further assessed or included in the study. Chemicals that were not discernible included PFPeA, perfluoropentanoic acid; PFHxA, perfluorohexanoic acid; PFTrDA, perfluorotridecanoic acid; PFBS, perfluorobutanesulfonic acid; PFPeS, perfluoropentanesulfonic acid; PFHpS, perfluoroheptanesulfonic acid; PFDS, perfluorodecane sulfonic acid; 2cx-MEHP, 2-carboxymethyl hexyl phthalate; oxo-MiNP, 7-oxo monoisononyl phthalate; OH-MiNP, 7-hydroxy monoisononyl phthalate; OH-MPHP, hydroxy monoisopropyl phthalate; MCiNP, Mono(carboxyisooctyl) phthalate). Chemicals not achieving the LOD threshold (>75% of samples above the LOD) included PFDoDA, perfluorododecanoic acid; 5-oxo-MEHP, 5-oxo mono(2-ethylhexyl) phthalate; 5-OH-MEHP, 5-hydroxy mono(2-ethylhexyl) phthalate. PFDoDA was measured in both batches of maternal plasma samples with only 22% of measurements above the LOD. The LOD for PFDoDA was 0.02 ng/mL in batch 1 and 0.05 ng/mL in batch 2. We only measured 5-oxo-MEHP and 5-OH-MEHP in batch 1. The LOD was 0.1 ng/mL for 5-oxo-MEHP and only 49% was above the LOD. For 5-OH-MEHP, the LOD was 0.05 ng/mL and 74% was above the LOD.

The laboratory participated successfully in the HBM4EU QA/QC program for PFAS analysis and participates bi-annually in the German External Quality Assessment Scheme (G-EQUAS) coordinated by the University of Erlangen-Nuremberg, Germany, for PFOA, PFNA, PFDA, PFBS, PFHpS, PFHxS, and PFOS analysis in serum. In addition, the laboratory participated in G-EQUAS for analysis of phthalate metabolites and triclosan in urine.

**Figure S1.** Flowchart of the selection of the study population.


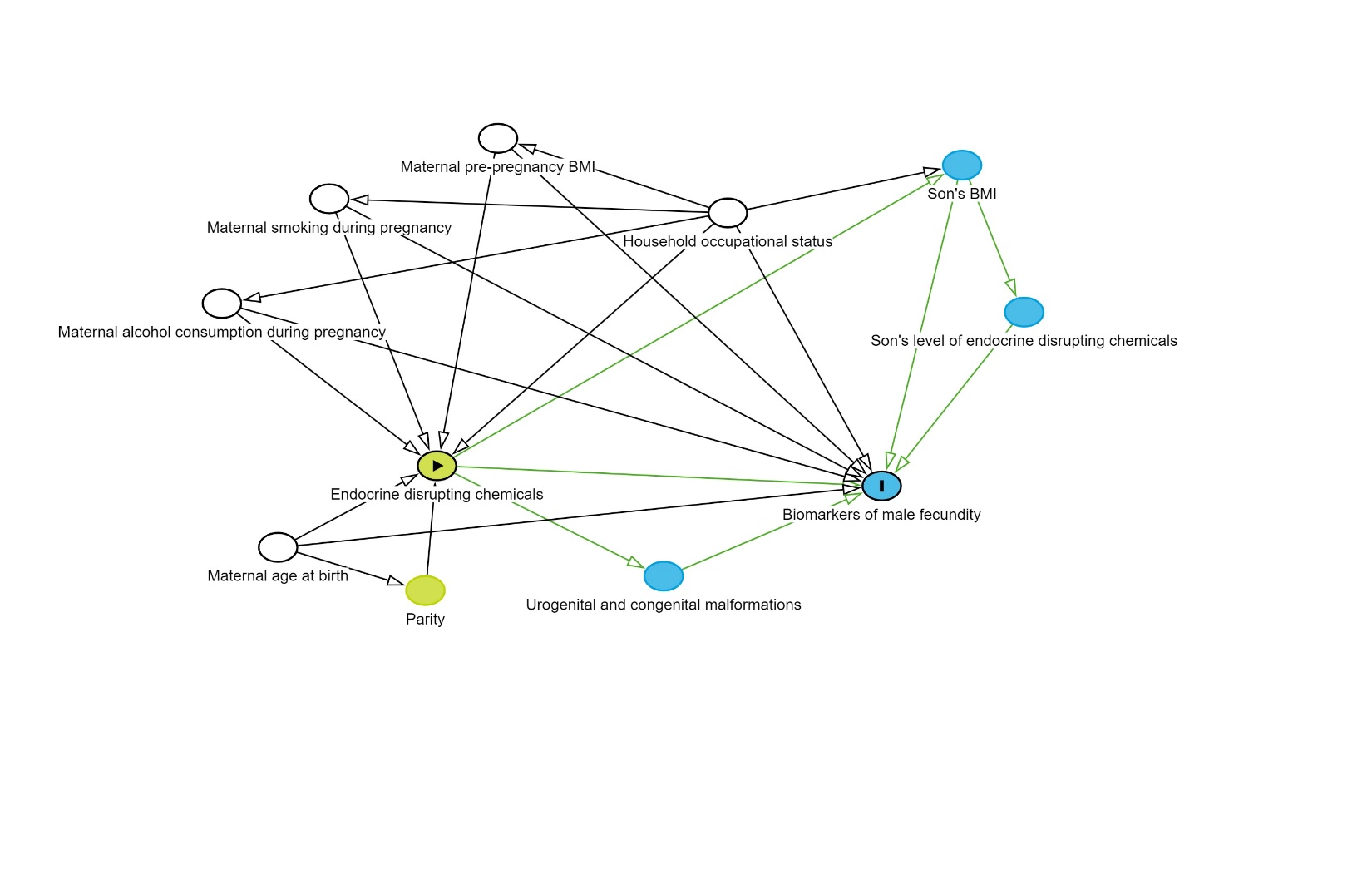


Excluded:
Insufficient amount of maternal plasma
for chemical analyses (N=188)
Undescended testicles in the scrotum (N=6)
Missing on covariates for analyses of fetal exposure to endocrine disrupting chemicals (N=23)

Sons born of women enrolled in the DNBC:

N=49,653

Not eligible:
Withdrawn from the DNBC (N=2,742)
No maternal participation in questionnaires (N=5,393)
No maternal blood sample (N=1,793)
Not fulfilling criteria of age or geography (N=18,102)

Not invited to participate in the FEPOS cohort (N=15,926)

Excluded:
Declined invitation or informed consent (N=4,450)
Did not answer FEPOS questionnaire (N=74)
Did not attend clinical examination (N=115)

Study participants included in this study:

N=841

Enrolled in the FEPOS cohort:

N=1,058

Invited to participate in the FEPOS cohort:

N=5,697

Eligible for inclusion in the FEPOS cohort:

N=21,623

**Figure S2**. Directed Acyclic Graph of the assumed relations between fetal exposure to a mixture of endocrine disrupting chemicals and biomarkers of male fecundity in young adulthood.

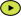
 Exposure;
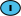
 Outcome;
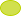
 Ancestor of exposure;
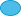
  Ancestor of outcome;
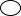
 Adjusted variable;
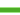
 Causal path.

| **Table S1.** Adjusted mixture coefficients and quadratic terms (95% confidence intervals) for associations between biomarkers of male fecundity and chemical mixtures measured in maternal plasma samples during pregnancy. | | | | | | | |
| --- | --- | --- | --- | --- | --- | --- | --- |
|  |  | **Mean difference (**ψ**) (95% CI)** | | | | | |
|  |  | **Overall mixture** | | **PFAS mixture** | | **Non-persistent chemical mixture** | |
| **Biomarkers of male fecundity** | ***n*** | Mixture  coefficient | ^2 term | Mixture  coefficient | ^2 term | Mixture  coefficient | ^2 term |
| Sperm concentration (×10^6^ per mL) | 829 | -8.4 (-28.8, 12.0) | 1.4 (-5.3, 8.0) | -3.1 (-20.7, 14.6) | -0.4 (-6.2, 5.4) | -3.1 (-15.4, 9.3) | 1.1 (-2.9, 5.1) |
| Total sperm count (×10^6^) | 688 | -9.7 (-78.5, 59.0) | -2.0 (-24.0, 20.0) | -21.3 (-86.2, 43.5) | 1.9 (-19.5, 23.3) | 16.3 (-29.7, 62.4) | -5.6 (-20.5, 9.3) |
| Morphologically normal sperm (%) | 813 | -2.5 (-5.0, 0.1) | 0.7 (-0.1, 1.5) | -1.1 (-3.2, 1.1) | 0.2 (-0.5, 1.0) | -1.3 (-3.0, 0.4) | 0.5 (-0.1, 1.0) |
| Testosterone (nmol/L) | 828 | -0.2 (-3.3, 2.9) | 0.2 (-0.8, 1.2) | -0.8 (-3.4, 1.8) | 0.2 (-0.7, 1.0) | 0.9 (-1.0, 2.8) | -0.1 (-0.7, 0.5) |
| Estradiol (pmol/L) | 828 | -19.1 (-35.3, -2.8) | 6.0 (0.9, 11.1) | -12.6 (-25.2, 0.0) | 3.8 (-0.2, 7.8) | -5.9 (-16.2, 4.4) | 2.1 (-1.2, 5.4) |
| Average testicular volume (mL) | 836 | 0.1 (-2.7, 2.9) | -0.2 (-1.1, 0.7) | -0.2 (-2.4, 2.0) | 0.0 (-0.7, 0.7) | 0.1 (-1.8, 2.0) | -0.1 (-0.8, 0.5) |
|  |  | **Percent (%) difference (95% CI)** | | | | | |
| Semen volume (mL)* | 688 | 3 (-24, 41) | -2 (-11, 9) | -10 (-30, 14) | 3 (-5, 11) | 16 (-5, 42) | -5 (-11, 2) |
| Non-progressive and immotile sperm (%)* | 813 | 7 (-17, 39) | -1 (-9, 7) | 14 (-8, 41) | -2 (-9, 5) | -7 (-20, 9) | 1 (-4, 7) |
| DNA fragmentation index (%)* | 776 | -6 (-29, 26) | 2 (-8, 12) | 0 (-20, 25) | 0 (-7, 8) | -9 (-24, 10) | 2 (-4, 9) |
| FSH (IU/L)* | 827 | 37 (3, 81) | -8 (-16, 1) | 34 (7, 66) | -7 (-14, 0) | 1 (-18, 24) | 0 (-6, 7) |
| LH (IU/L)* | 827 | 6 (-14, 32) | -2 (-8, 6) | 14 (-3, 34) | -3 (-8, 2) | -7 (-20, 8) | 2 (-3, 7) |
| SHBG (nmol/L)* | 828 | 5 (-14, 28) | -2 (-8, 5) | 7 (-9, 25) | -3 (-8, 2) | 1 (-10, 15) | 0 (-4, 5) |
| Note: Associations were estimated using linear models in quantile g-computation including a quadratic term for all exposures within the mixture. Psi (ψ) is the mixture coefficient and represent the difference in biomarkers of male fecundity. Asterisk (*) indicates models where outcomes were natural log (ln)-transformed; effect estimates thus reflect percentage difference in the outcome per quartile increase in the mixture (calculated using the equation: ([exp(ψ)-1] × 100).  The joint effect of the non-linear analyses can be determined by the quadratic term coefficient (^2 term) as well as the mixture coefficient for the lower order joint effect, as for a traditional linear regression model.  Model adjustments and abbreviations can be seen in **Table 5.** | | | | | | | |

| **Table S2.** Adjusted change (95% confidence intervals) in biomarkers of male fecundity per one-quartile increase in all exposures within the overall chemical mixture and weights representing the proportion of positive or negative partial effects for each exposure biomarker using linear models in quantile g-computation models without bootstrapping. | | | | | | | | | | | | | | | | | | | | | | | | | | | | | | | |
| --- | --- | --- | --- | --- | --- | --- | --- | --- | --- | --- | --- | --- | --- | --- | --- | --- | --- | --- | --- | --- | --- | --- | --- | --- | --- | --- | --- | --- | --- | --- | --- |
|  |  |  | **Positive and negative weights** | | | | | | | | | | | | | | | | | | | | | | | | | | | | |
| **Biomarkers of male fecundity** | ***n*** | **Mean difference**  **(95% CI)** | **PFHxS** | | | **PFHpA** | | | **PFOA** | | | **PFOS** | | | **PFNA** | | | **PFDA** | | | **PFUnDA** | | | **Triclosan** | | | **5cx-MEPP** | | | **cx-MiNP** | |
|  |  |  | **Pos.** | **Neg.** | **Pos.** | | **Neg.** | **Pos.** | | **Neg.** | **Pos.** | | **Neg.** | **Pos.** | | **Neg.** | **Pos.** | | **Neg.** | **Pos.** | | **Neg.** | **Pos.** | | **Neg.** | **Pos.** | | **Neg.** | **Pos.** | | **Neg.** |
| Sperm concentration (×10^6^ per mL) | 829 | -4.0 (-9.5, 1.5) |  | 0.15 |  | | 0.26 |  | | 0.02 |  | | 0.14 | 0.57 | |  |  | | 0.24 | 0.07 | |  | 0.29 | |  | 0.06 | |  |  | | 0.19 |
| Total sperm count (×10^6^) | 688 | -16.1 (-33.5, 1.3) |  | 0.10 |  | | 0.25 |  | | 0.20 | 0.14 | |  | 0.40 | |  |  | | 0.28 | 0.19 | |  | 0.24 | |  | 0.09 | |  |  | | 0.17 |
| Morphologically normal sperm (%) | 813 | -0.3 (-1.0, 0.3) |  | 0.07 |  | | 0.25 |  | | 0.09 |  | | 0.06 | 0.64 | |  |  | | 0.48 | 0.28 | |  |  | | 0.01 | 0.08 | |  |  | | 0.04 |
| Average testicular volume (mL) | 836 | -0.5 (-1.2, 0.3) | 0.23 |  | 0.05 | |  |  | | 0.10 |  | | 0.43 | 0.64 | |  |  | | 0.05 |  | | 0.16 |  | | 0.04 | 0.08 | |  |  | | 0.23 |
| Testosterone (nmol/L) | 828 | 0.3 (-0.4, 1.0) |  | 0.03 |  | | 0.30 | 0.18 | |  | 0.14 | |  |  | | 0.35 |  | | 0.26 | 0.08 | |  | 0.26 | |  |  | | 0.06 | 0.32 | |  |
| Estradiol (pmol/L) | 828 | -1.2 (-5.0, 2.7) |  | 0.15 |  | | 0.08 | 0.48 | |  |  | | 0.12 |  | | 0.34 | 0.03 | |  | 0.04 | |  | 0.24 | |  |  | | 0.32 | 0.20 | |  |
|  |  | **Percent difference**  **(95% CI)** |  | | | | | | | | | | | | | | | | | | | | | | | | | | | | |
| Semen volume (mL)* | 688 | -3 (-9, 5) |  | 0.26 | 0.27 | |  |  | | 0.33 | 0.24 | |  |  | | 0.19 | 0.26 | |  | 0.26 | |  |  | | 0.00 | 0.24 | |  |  | | 0.08 |
| Non-progressive and immotile sperm (%)* | 813 | 5 (-1, 11) | 0.27 |  | 0.12 | |  | 0.08 | |  | 0.29 | |  |  | | 0.47 |  | | 0.12 |  | | 0.12 |  | | 0.38 |  | | 0.03 | 0.05 | |  |
| DNA fragmentation index (%)* | 776 | -2 (-8, 6) | 0.27 |  | 0.18 | |  | 0.07 | |  |  | | 0.17 |  | | 0.28 |  | | 0.22 |  | | 0.22 |  | | 0.21 | 0.21 | |  |  | | 0.12 |
| FSH (IU/L)* | 827 | 7 (0, 16) | 0.31 |  | 0.08 | |  |  | | 0.42 | 0.06 | |  | 0.16 | |  |  | | 0.16 |  | | 0.16 |  | | 0.42 | 0.24 | |  | 0.02 | |  |
| LH (IU/L)* | 827 | 1 (-4, 7) | 0.13 |  |  | | 0.24 | 0.44 | |  | 0.03 | |  |  | | 0.11 | 0.26 | |  | 0.26 | |  |  | | 0.33 |  | | 0.04 |  | | 0.28 |
| SHBG (nmol/L)* | 828 | 0 (-5, 5) | 0.00 |  |  | | 0.16 |  | | 0.05 | 0.29 | |  |  | | 0.48 |  | | 0.10 |  | | 0.10 | 0.32 | |  | 0.26 | |  |  | | 0.22 |
| Note: Asterisk (*) indicates models where outcomes were natural log (ln)-transformed; effect estimates thus reflect percentage difference in the outcome per quartile increase in the mixture (calculated using the equation: ([exp(ψ)-1] × 100).  The qgcomp package does not estimate weights when using bootstrapping, we thus examined weights as an exploratory sensitivity analyses using models similar to our primary models but without bootstrapping. Weights from the qgcomp models can be interpreted as the relative contributions of each chemical to either the positive (+) or negative (-) partial effects. The weights should not be interpreted as individual effect sizes as models were performed to estimate joint effects of an overall chemical mixture and because of the limited precision of the joint effect estimates.  Model adjustments and abbreviations can be seen in **Table 5.** | | | | | | | | | | | | | | | | | | | | | | | | | | | | | | | |
